# Air Pollution and Deaths among Elderly Residents of São Paulo, Brazil: An Analysis of Mortality Displacement

**DOI:** 10.1289/EHP98

**Published:** 2016-10-07

**Authors:** Amine Farias Costa, Gerard Hoek, Bert Brunekreef, Antônio C.M. Ponce de Leon

**Affiliations:** 1 Institute of Social Medicine, Rio de Janeiro State University, Rio de Janeiro, Brazil; 2 Brazilian National Cancer Institute, Rio de Janeiro, Brazil; 3 Institute for Risk Assessment Sciences, Utrecht University, Utrecht, the Netherlands; 4 Julius Center for Health Sciences and Primary Care, University Medical Center Utrecht, Utrecht, the Netherlands

## Abstract

**Background:**

Evaluation of short-term mortality displacement is essential to accurately estimate the impact of short-term air pollution exposure on public health.

**Objectives:**

We quantified mortality displacement by estimating single-day lag effects and cumulative effects of air pollutants on mortality using distributed lag models.

**Methods:**

We performed a daily time series of nonaccidental and cause-specific mortality among elderly residents of São Paulo, Brazil, between 2000 and 2011. Effects of particulate matter smaller than 10 μm (PM_10_), nitrogen dioxide (NO_2_) and carbon monoxide (CO) were estimated in Poisson generalized additive models. Single-day lag effects of air pollutant exposure were estimated for 0-, 1- and 2-day lags. Distributed lag models with lags of 0–10, 0–20 and 0–30 days were used to assess mortality displacement and potential cumulative exposure effects.

**Results:**

PM_10_, NO_2_ and CO were significantly associated with nonaccidental and cause-specific deaths in both single-day lag and cumulative lag models. Cumulative effect estimates for 0–10 days were larger than estimates for single-day lags. Cumulative effect estimates for 0–30 days were essentially zero for nonaccidental and circulatory deaths but remained elevated for respiratory and cancer deaths.

**Conclusions:**

We found evidence of mortality displacement within 30 days for nonaccidental and circulatory deaths in elderly residents of São Paulo. We did not find evidence of mortality displacement within 30 days for respiratory or cancer deaths.

**Citation:**

Costa AF, Hoek G, Brunekreef B, Ponce de Leon AC. 2017. Air pollution and deaths among elderly residents of São Paulo, Brazil: an analysis of mortality displacement. Environ Health Perspect 125:349–354; http://dx.doi.org/10.1289/EHP98

## Introduction

Short-term exposure to air pollution has been associated with a variety of adverse health effects, such as overall, circulatory, and respiratory mortality [Anderson et al. 2007; World Health Organization (WHO) 2006, 2013].

It has been suggested that short-term exposure to air pollution only affects a frail subpopulation with an elevated risk of dying owing to its poor health conditions. Consequently, an air pollution episode could deplete this frail group and advance deaths among some by a limited number of days or weeks, followed by a period with a mortality rate that is lower than expected. This phenomenon is known as “mortality displacement” or “harvesting effect” (Schwartz 2000a; Zeger et al. 1999). It is important to identify mortality displacement for public health reasons. If air pollution–related deaths are displaced only by a few days, the public health impact measured in loss of life expectancy would be less than when deaths are brought forward by a much greater period of time (Schwartz 2000a).

Because the effects of air pollution on mortality could occur on the same day of exposure or on later days, the so-called lag structures need to be investigated to quantify these effects. However, if multiple lags are used in one model, common regression models will be susceptible to collinearity problems because of the high correlation among exposures on consecutive days. The solution for this problem was the development of distributed lag models (DLMs) using smooth functions, such as polynomials, to describe the relationship between lagged exposure of multiple days (Gasparrini et al. 2010; Zanobetti et al. 2000). Recently, the investigation of lag structures has been improved through the use of DLMs by several authors in different study types (Armstrong 2006; Gasparrini et al. 2010; Gasparrini 2014; Roberts and Martin 2007; Samoli et al. 2013; Schwartz 2000b). In some time series studies, the DLM was applied to quantify a cumulative effect over multiple lagged days (Braga et al. 2001; Filleul et al. 2004; O’Neill et al. 2008; Romieu et al. 2012; Samoli et al. 2009, 2013; Schwartz 2000b), to evaluate the mortality displacement attributable to air pollution (Goodman et al. 2004; Zanobetti et al. 2000, 2002, 2003; Zanobetti and Schwartz 2008), or for a combination of both purposes.

In most cases, studies that used DLM with lag structures ≤ 60 days have not found mortality displacement within this time period, which suggests that the effects of air pollution on mortality are not simply because of deaths being advanced by a few days or weeks. In these studies, effects based on single-day lags underestimate cumulative effects (Goodman et al. 2004; Zanobetti et al. 2002, 2003; Zanobetti and Schwartz 2008). A European multicity study (Zanobetti et al. 2002, 2003) estimated the effects of a 10 μg/m^3^ increase in particulate matter < 10 μm (PM_10_) and reported that nonaccidental, cardiovascular, and respiratory deaths increased 1.61% [95% confidence interval (95% CI): 1.02, 2.20], 1.97% (95% CI: 1.38, 2.55), and 4.20% (95% CI: 1.08, 7.42), respectively, for cumulative effects ≤ 40 days. In contrast, corresponding estimates for average exposures on the same day and on the previous day were only 0.70% (95% CI: 0.43, 0.97), 0.69% (95% CI: 0.31, 1.08), and 0.74% (95% CI: –0.17, 1.66), respectively.

Mortality displacement in U.S. and European populations may differ from displacement in Latin America because of differences in population, health care, and other characteristics. However, to our knowledge, no studies of mortality displacement have been reported based on data from Latin America. In addition, we are not aware of studies that have analyzed mortality displacement in relation to daily time series of nitrogen dioxide (NO_2_) and carbon monoxide (CO) exposures.

São Paulo is the largest and most developed city in Brazil, and it is also one of the most polluted cities (IEMA 2014). Recently, the Multicity Study of Air Pollution and Mortality in Latin America (ESCALA; Estudio de Salud y Contaminación del Aire en Latinoamérica) (Romieu et al. 2012), reported significant associations between mortality in São Paulo and single-day lagged exposures and short cumulative-lag exposures (≤ 3 days) to PM_10_, encouraging further investigation of these data.

The aim of the present study was to investigate evidence for mortality displacement in the association between air pollution and daily mortality among elderly residents of São Paulo, Brazil.

## Methods

We conducted a daily time series study of the relationships between nonaccidental deaths and PM_10_, NO_2_, and CO exposures. The analysis included deaths that occurred among elderly residents (≥ 60 years) of São Paulo, Brazil, between 2000 and 2011.

Numbers of daily deaths (in men and women combined) were obtained from the records kept in the Mortality Information System of the Brazilian Public Health System [Departamento de Informática do SUS (DATASUS) 2014] and reviewed by the Improvement Program for Mortality Information of the Health Secretariat of São Paulo City [Secretaria Municipal da Saúde da Prefeitura de São Paulo (SMSSP) 2014]. Outcomes were classified according to the WHO’s *International Classification of Diseases and Related Health Problems* (10th revision) as all non-accidental causes (all groups except S00 to T98 and V01 to Y98), circulatory diseases (group I), cerebrovascular diseases (codes I60 to I69), respiratory diseases (group J), chronic lower respiratory diseases (codes J40 to J47), and cancer (groups C and D, until code D48).

Air pollutant data were obtained from records provided by the Environmental Company of the State of São Paulo [Companhia Ambiental do Estado de São Paulo (CETESB) 2014]. PM_10_ was measured using beta radiation (10 possible monitors), NO_2_ was measured using chemiluminescence (7 possible monitors) and CO with nondispersive infrared sensing (9 possible monitors). Measurements were considered valid if ≥ 16 hourly measurements were collected during each day for each site. Only sites with ≥ 16 hourly measurements for each day, and with < 25% missing data for the whole period, were used in the analysis. In addition, measurements at each site had to be highly correlated with those at other sites (Spearman correlation ≥ 0.80) (see Table S1) for a site to be included, leaving 3 sites for PM_10_ and CO (24-hr average and maximum 8-hr moving average, respectively) and 2 sites for NO_2_ (24-hr average) (see Table S2). Missing data for each site were imputed based on linear regressions using data for the same pollutant measured at other sites at the same time. Remaining missing air pollutant data were estimated by application of a cubic smoothed spline with 4 degrees of freedom (df) per year; the *mtsdi* package (Multivariate Time Series Data Imputation) (Junger and Ponce de Leon 2012) for R (version 3.1.2; R Project for Statistical Computing) was used for this purpose (see Table S2).

After imputation, daily city levels were calculated for each pollutant by averaging all available data across the selected monitoring sites. Daily averages for temperature and relative humidity were obtained from measurements performed by the Brazilian Institute of Meteorology (one site) [Instituto Nacional de Meteorologia (INMET) 2014] and by the Airspace Control Institute of the Brazilian Defense Ministry (two sites) [Instituto do Controle do Espaço Aéreo (ICEA) 2014].

The Poisson generalized additive model (GAM) was fitted to estimate single-day lag effects of air pollutant exposures on lag 0, lag 1, and lag 2 days. In addition, the Poisson generalized additive DLM was fitted for lags ≤ 30 days for nonaccidental and specific causes of death and for lags ≤ 40 days for nonaccidental deaths (the latter to compare with previous studies of total mortality).

For the DLM analysis, we used a matrix of second-degree polynomials to estimate separate effects for cumulative lags of 0–10, 0–20, 0–30, and 0–40 days. A polynomial structure was used to fit a smooth shape for these effects because it has been shown to reduce noise and bias compared with an unconstrained DLM (Schwartz 2000b; Zanobetti et al. 2002).

All Poisson regression analyses were performed in R using the *mgcv* (Mixed GAM Computation Vehicle with GCV/AIC/REML smoothness) (Wood 2011) and *dlnm* (Distributed Lag Non-linear Models) (Gasparrini 2011) packages.

Models were adjusted using a thin plate regression spline for temporal trend and seasonality (4–7 df per year), mean daily temperature at single-day lags of 3 or 5 days (2 or 3 df), and mean daily relative humidity at single-day lag 3 (2 or 3 df), following the methodology used by the ESCALA project (Romieu et al. 2012). The choice of the most appropriate lags for temperature and relative humidity as well as the number of degrees of freedom for these variables and for temporal trend and seasonality were based on the Akaike Information Criterion from each model. Categorical variables for weekdays and holidays were also included. Model adjustments were different for each outcome (see Table S3).

Residual diagnostics were conducted by analyzing *a*) scatter plots of deviance residuals for variation around the long-term pattern, *b*) partial autocorrelation functions for residual autocorrelation and overfitting, *c*) periodograms for residual seasonality, and *d*) Q-Q plots for normality of standardized deviance residuals.

Mortality displacement was assessed from the shape of the association between air pollutants and deaths. When mortality displacement is within the time window analyzed, one expects a drop in effects for longer lags, making them negative until the frail subpopulation pool is replenished. In cumulative effects, mortality displacement results in a decrease towards zero (the effects cancel out or partially cancel out), and it is expected that the confidence interval for the sum of the relative risks includes 1 (Schwartz 2000a; Zanobetti et al. 2000). The air pollution effect estimates are presented as an increase or a decrease in the percentage of deaths, and in their 95% CIs, for 10-μg/m^3^ increases in PM_10_ and NO_2_ and for a 1-ppm increase in CO.

A sensitivity analysis was performed using unconstrained DLM ≤ 30 days, that is, without a polynomial structure to specify the relationship between effects and lagged days, for PM_10_, NO_2_, and CO. We performed separate PM_10_ analyses to evaluate the potential influence of the degrees of freedom specified for the trend variable (specifically, 4 vs. 6 df/year for nonaccidental mortality, 5 vs. 6 df/year for circulatory mortality, 6 vs. 5 df/year for respiratory and chronic respiratory mortality, and 5 vs. 4 df/year for cerebrovascular and cancer mortality). In addition, we estimated the effects of PM_10_ on deaths among residents of all ages because PM_10_ would be expected to have a limited impact on an analysis of mortality. Finally, we repeated the PM_10_ analyses adjusting for mean temperature over a 0–10 day lag using exposure–response and lag–response curves according to Gasparrini et al. (2015) instead of the single-day lags of 3 or 5 days used in the primary models.

All analyses were performed using R software, version 3.1.2 (R Project for Statistical Computing). Statistical significance was set at *p*-value < 0.05.

## Results

The mean concentrations of PM_10_, NO_2_, and CO for the whole period of analysis were 40.8 μg/m^3^, 51.1 μg/m^3^ and 1.6 ppm, respectively (Table 1). The mean temperature ranged from 8.9°C to 28.9°C, and the mean relative humidity ranged from 27.3% to 94.3%. Among all elderly persons, there were 109 deaths per day on average, of which 45 were attributed to circulatory diseases, 17 attributed to respiratory diseases, and 23 attributed to cancer.

**Table 1:** Daily summary statistics of air pollutants, weather variables and number of deaths among elderly.

Environment variable/death	Daily measures					
	Minimum	1st Quartile	Median	Mean	3rd Quartile	Maximum
Environment variables
PM_10_ (μg/m^3^)	4.6	26.5	37.1	40.8	50.4	158.7
NO_2_ (μg/m^3^)	9.2	38.4	48.2	51.1	60.9	150.5
CO (ppm)	0.4	1.0	1.4	1.6	2.0	8.6
Mean temperature (ºC)	8.9	18.5	21.1	20.8	23.4	28.9
Mean relative humidity (%)	27.3	66.9	74.0	72.7	79.9	94.3
Deaths (*n*)
Nonaccidental	59.0	96.0	108.0	109.1	121.0	178.0
Circulatory	19.0	39.0	45.0	45.2	51.0	84.0
Cerebrovascular	1.0	9.0	11.0	11.5	14.0	28.0
Respiratory	3.0	13.0	17.0	17.0	20.0	42.0
Chronic respiratory	0.0	4.0	6.0	5.9	7.0	18.0
Cancer	7.0	19.0	23.0	22.9	26.0	46.0
Notes: CO, carbon monoxide; NO_2_, nitrogen dioxide; PM_10_, particulate matter smaller than 10 μm.						

PM_10_, NO_2_, and CO exposures were associated with nonaccidental deaths and with all specific causes of death evaluated (Table 2). Considering nonaccidental deaths, the estimated daily increases were 0.37% (95% CI: 0.20, 0.55), 0.40% (95% CI: 0.21, 0.60), and 1.07% (95% CI: 0.68, 1.47) related to PM_10_, NO_2_, and CO exposure, respectively, on lag 0.

**Table 2:** Percent change (95% confidence interval)*^a^* in number of deaths associated with air pollutant levels for different single-day lags.*^b^*

Deaths	Percent change (95% confidence interval)		
	Lag 0	Lag 1	Lag 2
PM_10_
Nonaccidental	0.37 (0.20, 0.55)	0.54 (0.36, 0.72)	0.60 (0.40, 0.80)
Circulatory	0.01 (–0.27, 0.29)	0.16 (–0.14, 0.47)	0.40 (0.07, 0.73)
Cerebrovascular	1.60 (0.99, 2.22)	1.44 (0.74, 2.15)	0.04 (–0.58, 0.66)
Respiratory	1.33 (0.83, 1.83)	0.98 (0.41, 1.56)	0.61 (0.11, 1.12)
Chronic respiratory	1.43 (0.58, 2.27)	1.15 (0.18, 2.12)	0.56 (–0.29, 1.41)
Cancer	0.71 (0.27, 1.15)	0.55 (0.04, 1.06)	0.09 (–0.35, 0.54)
NO_2_
Nonaccidental	0.40 (0.21, 0.60)	0.50 (0.31, 0.70)	0.58 (0.38, 0.79)
Circulatory	0.31 (0.01, 0.61)	0.42 (0.11, 0.74)	0.55 (0.22, 0.89)
Cerebrovascular	1.51 (0.88, 2.14)	1.32 (0.63, 2.02)	0.17 (–0.48, 0.83)
Respiratory	1.22 (0.71, 1.74)	0.67 (0.10, 1.24)	0.41 (–0.12, 0.95)
Chronic respiratory	1.21 (0.34, 2.08)	0.74 (–0.22, 1.71)	0.49 (–0.42, 1.4)
Cancer	0.93 (0.48, 1.38)	0.48 (–0.01, 0.98)	0.02 (–0.45, 0.49)
CO
Nonaccidental	1.07 (0.68, 1.47)	1.04 (0.64, 1.44)	0.74 (0.32, 1.17)
Circulatory	0.84 (0.23, 1.45)	0.28 (–0.35, 0.92)	–0.14 (–0.78, 0.50)
Cerebrovascular	2.39 (1.08, 3.71)	0.99 (–0.38, 2.39)	0.31 (–0.97, 1.59)
Respiratory	2.54 (1.47, 3.63)	1.66 (0.52, 2.80)	0.72 (–0.33, 1.78)
Chronic respiratory	2.19 (0.41, 4.01)	1.87 (–0.02, 3.79)	1.35 (–0.41, 3.13)
Cancer	1.40 (0.46, 2.35)	0.16 (–0.83, 1.16)	–0.29 (–1.21, 0.63)
Notes: CO, carbon monoxide; NO_2_, nitrogen dioxide; PM_10_, particulate matter smaller than 10 μm. ^***a***^Associated with a 10-μg/m^3^ increase in PM_10_ and NO_2_ and with a 1-ppm increase in CO. ^***b***^Results from a Poisson generalized additive model using single-day lag structures for PM_10_, NO_2_, and CO, adjusted by trend, seasonality, temperature, relative humidity, weekdays, and holidays.			

We estimated significant cumulative effects for lag 0–10 for most causes of death and pollutants that were substantially larger than the estimates for the single lags (Table 3). Cumulative effect estimates for lag 0–20 remained significant for nonaccidental deaths associated with PM_10_ and NO_2_ and for respiratory and cancer deaths associated with all pollutants. The cumulative effect estimates for lag 0–30 remained high and significant for respiratory and cancer deaths associated with PM_10_ and NO_2_. Cumulative effect estimates for nonaccidental or cerebrovascular mortality at lag 0–30 were not statistically significant for any pollutant and, in general, were close to the null, but associations with circulatory deaths were negative and statistically significant for NO_2_ and CO and were nearly significant for PM_10_.

**Table 3:** Cumulative percent change (95% confidence interval)*^a^* in number of deaths associated with air pollutant levels for different cumulative lag structures.*^b^*

Deaths	Percent change (95% confidence interval)		
	Lag 0 to 10	Lag 0 to 20	Lag 0 to 30
PM_10_
Nonaccidental	1.22 (0.84, 1.60)	0.88 (0.33, 1.44)	–0.10 (–0.82, 0.62)
Circulatory	0.72 (0.11, 1.33)	0.04 (–0.85, 0.93)	–1.11 (–2.21, 0.01)
Cerebrovascular	2.33 (1.14, 3.54)	1.05 (–0.58, 2.71)	0.72 (–1.39, 2.87)
Respiratory	3.40 (2.45, 4.36)	3.45 (2.05, 4.87)	2.81 (0.99, 4.66)
Chronic respiratory	2.95 (1.34, 4.57)	1.58 (–0.75, 3.96)	2.41 (–0.66, 5.57)
Cancer	1.55 (0.75, 2.43)	1.59 (0.50, 2.68)	1.98 (0.58, 3.40)
NO_2_
Nonaccidental	1.47 (1.05, 1.89)	1.15 (0.52, 1.78)	0.22 (–0.60, 1.05)
Circulatory	1.34 (0.66, 2.02)	0.55 (–0.46, 1.57)	–1.45 (–2.77, –0.12)
Cerebrovascular	1.99 (0.69, 3.30)	0.62 (–1.20, 2.47)	–0.14 (–2.47, 2.24)
Respiratory	3.18 (2.13, 4.25)	3.15 (1.58, 4.76)	2.98 (0.94, 5.06)
Chronic respiratory	2.83 (1.03, 4.66)	0.83 (–1.81, 3.54)	1.34 (–2.09, 4.90)
Cancer	1.84 (0.93, 2.76)	2.38 (1.05, 3.72)	2.35 (0.67, 4.06)
CO
Nonaccidental	2.01 (1.05, 2.97)	0.41 (–0.99, 1.83)	–1.47 (–3.29, 0.39)
Circulatory	1.18 (–0.30, 2.68)	–0.11 (–3.29, 1.11)	–4.22 (–7.05, –1.30)
Cerebrovascular	3.54 (0.70, 6.45)	0.88 (–3.09, 5.01)	0.34 (–4.78, 5.74)
Respiratory	6.41 (3.96, 8.92)	5.96 (3.36, 9.69)	3.21 (–1.32, 7.94)
Chronic respiratory	5.39 (1.37, 9.57)	1.81 (–3.91, 7.86)	2.82 (–4.59, 10.80)
Cancer	1.36 (–0.64, 3.41)	3.50 (0.50, 6.58)	2.85 (–0.96, 6.81)
Notes: CO, carbon monoxide; NO_2_, nitrogen dioxide; PM_10_, particulate matter smaller than 10 μm. ^***a***^Associated with a 10-μg/m^3^ increase in PM_10_ and NO_2_ and with a 1-ppm increase in CO. ^***b***^Results from a Poisson generalized additive distributed lag model, constrained with a second degree polynomial, using cumulative lag structures of lags 0–10, 0–20, and 0–30 days for PM_10_, NO_2_, and CO, adjusted by trend, seasonality, temperature, relative humidity, weekdays, and holidays.			

**Figure 1:**
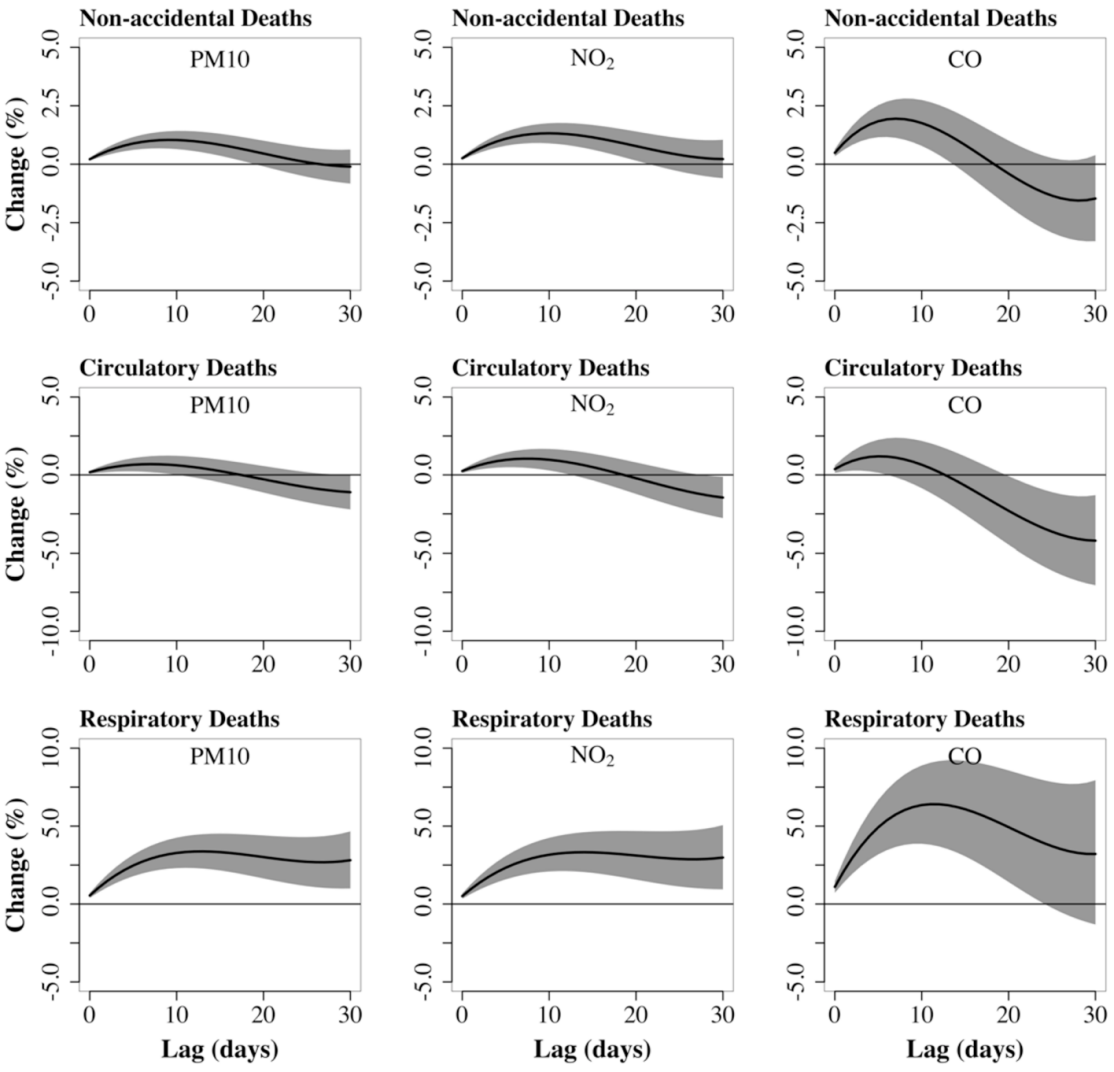
Cumulative percent change*^a^* in number of deaths associated with air pollutant levels of lag 0–30 days.*^b^* ***^a^***Associated with a 10-μg/m^3^ increase in particulate matter smaller than 10 μm (PM_10_) and nitrogen dioxide (NO_2_) and with a 1-ppm increase in carbon monoxide (CO). ***^b^***Results from a Poisson generalized additive distributed lag model, constrained with a second degree polynomial, using cumulative lag structures of lags 0–30 days for PM_10_, NO_2_, and CO, adjusted by trend, seasonality, temperature, relative humidity, weekdays, and holidays. The shadow area represents the 95% confidence interval.

Cumulative effect estimates for nonaccidental deaths increased and then decreased, becoming zero at 27 days for PM_10_, at 19 days for CO (Figure 1), and at 32 days for NO_2_ (not shown). When the analysis was extended to lag 0–40, the estimated effects at 40 days were similar to those at 30 days [0.00% (95% CI: –0.92, 0.92) for PM_10_, –0.13% (95% CI: –1.16, 0.91) for NO_2_, and –3.03% (95%CI: –5.37, –0.64) for CO]. The pattern was similar for circulatory deaths, with effect estimates crossing the null at 18, 19, and 13 days for PM_10_, NO_2_, and CO, respectively (Figure 1). In contrast, cumulative effect estimates for respiratory deaths remained positive throughout the 0–30 day lag. Effect estimates for single-day lagged exposures also supported mortality displacement for nonaccidental and circulatory deaths, with significant negative associations estimated for exposures lagged by approximately 10–27 days (see Figure S1).

The effect estimates and the pattern in the effect estimates in unconstrained DLM were similar to those in polynomial DLM (Table 4). The pattern of cumulative effect estimates for PM_10_ lagged 0–30 days was similar to the main analysis when we modeled the time trend using different degrees of freedom (see Table S4) and when we included deaths among residents of all ages instead of limiting the analysis to residents ≥ 60 years of age (see Table S5). Additionally, cumulative effect estimates for PM_10_ lagged 0–30 days were consistent with the primary analysis when we adjusted for daily temperature using a 0- to 10-day cumulative lag (see Table S6).

**Table 4:** Cumulative percent change (95% confidence interval)*^a^* in number of deaths, of lag 0–30 days, associated with air pollutant levels from an unconstrained model.*^b^*

Deaths	Percent change (95% confidence interval)		
	PM_10_	NO_2_	CO
Nonaccidental	–0.03 (–0.77, 0.71)	0.32 (–0.51, 1.16)	–1.40 (–3.24, 0.48)
Circulatory	–0.98 (–2.10, 0.16)	–1.30 (–2.64, 0.05)	–3.91 (–6.78, –0.94)
Cerebrovascular	0.45 (–1.68, 2.63)	–0.40 (–2.75, 2.00)	1.05 (–4.16, 6.54)
Respiratory	2.82 (0.97, 4.71)	3.02 (0.95, 5.24)	3.31 (–1.26, 8.09)
Chronic respiratory	2.04 (–1.07, 5.24)	0.98 (–2.49, 4.56)	2.72 (–4.74, 1.08)
Cancer	1.98 (0.57, 3.41)	2.53 (0.83, 4.26)	3.15 (–0.70, 7.15)
Notes: CO, carbon monoxide; NO_2_, nitrogen dioxide; PM_10_, particulate matter smaller than 10 μm. ^***a***^Associated with a 10-μg/m^3^ increase in PM_10_ and NO_2_ and with a 1-ppm increase in CO. ^***b***^Results from an unconstrained Poisson generalized additive distributed lag model using cumulative lag structures of lags 0–10, 0–20, and 0–30 days for PM_10_, NO_2_, and CO, adjusted by trend, seasonality, temperature, relative humidity, weekdays, and holidays.			

## Discussion

Few time series studies have addressed mortality displacement and cumulative effects of air pollution for periods of 1 month. Our findings suggest that in elderly residents of São Paulo, nonaccidental mortality was displaced by < 30 days by the three pollutants studied. However, the displacement patterns varied considerably for specific causes of death.

### Mortality Displacement

To our knowledge, this is the first study that assessed mortality displacement by air pollution using Brazilian data. São Paulo is a city with both a large population and a long continuous record of daily air pollution monitoring, making this city very suitable for the analysis we performed. Our findings provide evidence of mortality displacement for nonaccidental and circulatory deaths within 30 days, in contrast with other studies of this phenomenon.

One of the first DLM studies analyzed total suspended particulate (TSP) and mortality data from Milan, Italy between 1980 and 1989 (Zanobetti et al. 2000). The authors found a significant increase in total mortality ≤ 45 days (Table 5). Zanobetti et al. (2002, 2003) also analyzed mortality displacement related to PM_10_ levels in Europe between 1990 and 1997. The authors did not find evidence of the phenomenon within 40 days for all deaths (Zanobetti et al. 2002) or for circulatory deaths (Zanobetti et al. 2003) based on a meta-analysis of 10 cities (Table 5). Similarly, Goodman et al. (2004) found no evidence of mortality displacement within 40 days for all, circulatory, and respiratory deaths related to black smoke (BS) in Dublin, Ireland between 1980 and 1996 (Table 5). Furthermore, previous studies of TSP and deaths in Philadelphia, Pennsylvania from 1974 to 1988 (Zeger et al. 1999) and of particulate matter < 2.5 μm (PM_2.5_) and circulatory deaths in the Boston, Massachusetts area from 1979 to 1986 (Schwartz 2000a) did not report findings consistent with mortality displacement.

**Table 5:** Cumulative percent change (95% confidence interval) in number of nonaccidental, circulatory, and respiratory deaths by 10 μg/m^3^ increase in particulate air pollution among studies.

Study	Age group	Air pollutant	Period	Percent change^*a*^ (95% confidence interval)	
				Cumulative	Single lag
Non-accidental deaths
São Paulo^*b*^ (this study)	Elderly	PM_10_	Lag 0 to 40	0.00 (–0.92, 0.92)	0.37 (0.20, 0.55)^*c*^
Milan (Zanobetti et al. 2000)^*b*^	All ages	TSP	Lag 0 to 45	6.70 (3.80, 9.60)	2.20 (1.40, 3.10)
Boston (Schwartz 2000a)^*d*^	All ages	PM_2.5_	60-day window	3.75 (3.20, 4.30)	2.10 (1.50, 4.30)
10 European Cities (Zanobetti et al. 2002)^*b*^	All ages	PM_10_	Lag 0 to 40	1.57 (0.26, 2.88)	0.70 (0.43, 0.97)
Dublin (Goodman et al. 2004)^*b*^	All ages	BS	Lag 0 to 40	1.10 (0.80, 1.30)	0.40 (0.30, 0.60)
Circulatory deaths
São Paulo^*b*^ (this study)	Elderly	PM_10_	Lag 0 to 30	–1.11 (–2.21, 0.01)	0.01 (–0.27, 0.29)^*e*^
10 European Cities (Zanobetti et al. 2003)^*e*^	All ages	PM_10_	Lag 0 to 40	1.72 (1.20, 2.25)	0.69 (0.31, 1.08)
Dublin (Goodman et al. 2004)^*b*^	All ages	BS	Lag 0 to 40	1.10 (0.70, 1.50)	0.40 (0.20, 0.70)
Respiratory deaths
São Paulo^*b*^ (this study)	Elderly	PM_10_	Lag 0 to 30	2.81 (0.99, 4.66)	1.33 (0.83, 1.83)^*e*^
10 European Cities (Zanobetti et al. 2003)^*e*^	All ages	PM_10_	Lag 0 to 40	2.62 (0.19, 5.11)	0.74 (–0.17, 1.66)
Dublin (Goodman et al. 2004)^*b*^	All ages	BS	Lag 0 to 40	3.60 (3.00, 4.30)	0.20 (0.00, 0.50)
Notes: BS, black smoke; PM_2.5_, particulate matter smaller than 2.5 μm; PM_10_, particulate matter smaller than 10 μm; TSP, total suspended particulate. ^***a***^Interquartile range increase in Zanobetti et al. (2000) and 10-μg/m^3^ increase in other studies. ^***b***^Distributed lag model constrained with polynomial structure. ^***c***^Lag 0. ^***d***^STL (standard template library) algorithms with LOESS (locally weighted smoothing). ^***e***^Unconstrained distributed lag model.					

Disease-specific mortality and morbidity patterns, as well as prevention and treatment policies, differ between Brazil and other countries. These factors may contribute to differences in frailty compared with other populations and might explain the evidence of mortality displacement in our Brazilian study population. One example illustrating these differences is the study carried out between 1996 and 2001 by Martins et al. (2006) using DLM until lag 20. The authors found evidence of short-term displacement of circulatory hospital admissions among the elderly in São Paulo at cumulative lags of a few days only for PM_10_ and sulfur dioxide (SO_2_).

According to WHO, Brazil has higher mortality rates and Disability Adjusted Life Years because of circulatory diseases and a poorer risk factor profile, such as a greater hypertension prevalence, than countries in Europe or North America (WHO 2011). Additionally, as stated by WHO, Brazil has a low expenditure per capita on health and high income inequality; therefore, prevention and treatment of circulatory disease, and of other diseases, is likely not as good as those in high-income countries (WHO 2011).

Brazil has a national and free health care system with several specific policies for some diseases and risk factors. However, the low coverage by and complicated access to the system likely contribute to the poor health status of many inhabitants. In São Paulo, the primary health care system covered only 30.4% of the population in December of 2011, the last month evaluated in this study [Departamento de Atenção Básica (DAB) 2015]. According to a national survey carried out in 2013 [Instituto Brasileiro de Geografia e Estatística (IBGE) 2015], 39.0% of residences were registered in primary health care units in São Paulo, and only 35.0% of these were visited by primary health care teams.

In contrast with nonaccidental and circulatory mortality, we did not find evidence of mortality displacement within 30 days for respiratory or cancer mortality. This finding suggests that cumulative air pollution exposure may shift mortality away from circulatory diseases to other causes of death. We have no ready explanation for this phenomenon. The patterns differed for different causes of death, and noncausal explanations are also possible. Zanobetti et al. (2003) did not find evidence of mortality displacement for respiratory deaths related to PM_10_ exposure in a European multicity study (Table 5). Schwartz (2000a) found no indication for mortality displacement for cardiovascular mortality, whereas some indication was found for pneumonia and chronic obstructive pulmonary disease mortality.

Air pollution can influence the frail subpopulation in at least three ways: by increasing the mortality rate, by increasing recruitment into the group, and by delaying the recovery rate of the group. If only the mortality rate increases, mortality displacement can likely be observed. However, if air pollution affects all three mechanisms and increases the frail subpopulation size, larger positive associations between air pollution and mortality may be observed over increasing time intervals (Zanobetti and Schwartz 2008).

Alternatively, deaths from respiratory diseases and cancer may not represent responses to air pollution as acute as deaths from circulatory diseases (Zanobetti et al. 2003). This may explain the absence of observable mortality displacement over the 30-day time window analyzed in the present study.

It is difficult to disentangle the effects of temperature and air pollution. Although adjusting for temperature over longer lags (0–10 days) did not materially alter the evidence in support of mortality displacement (see Table S6), cumulative effects for some outcomes did change slightly, and we could not adjust for temperature over longer lag periods because of unstable estimates and problems with model convergence.

### Associations with Single Short Lags

Single-day lag and cumulative effects ≤ 5 days indicated significant increases in deaths associated with air pollutant levels, which is in line with results presented in other studies from São Paulo and in many studies throughout the world (see Tables S7 and S8). Associations between a 10-μg/m^3^ increase and overall and specific causes of deaths in São Paulo City reported by the ESCALA study (Romieu et al. 2012) were comparable to our estimates (see Table S7).

Cancer deaths were included in the present study because it is reasonable to think that individuals who have advanced cancer are also susceptible to air pollution episodes. Because short-term exposure to air pollution is not associated with carcinogenesis, the immediate cause of death is likely cardiovascular or respiratory, but the underlying cause noted on the death certificate will still be cancer. Positive associations were also reported between cancer deaths and TSP (Villeneuve et al. 2003), NO_2_, SO_2_, and O_3_ (Goldberg et al. 2013) in time series studies.

### Cumulative Effects ≤ 10 days

Our estimates of cumulative effects over 0–10 days were stronger than estimated effects for single-day lagged exposures, and the cumulative effect estimates remained significant up to lag 10 for all exposures and outcomes except for associations between CO and circulatory and cancer mortality. ESCALA (Romieu et al. 2012) estimated significant cumulative effects for PM_10_ exposure until lag 3 for São Paulo City, which were also stronger than effect estimates for single-day lags (see Table S8). Consistent with previous analyses that used DLM (Zanobetti et al. 2002, 2003) and other cumulative models (Schwartz 2000a), our findings suggest that relative risks may be underestimated when based on single-day lag effects.

## Conclusion

We found evidence of short-term mortality displacement for nonaccidental and circulatory deaths in an elderly Brazilian population. No evidence for mortality displacement within 30 days was found for respiratory and cancer deaths. Additional research is needed to confirm our findings and to identify potential mechanisms to explain them.

## Supplemental Material

(333 KB) PDF

## References

[r1] Anderson HR, Atkinson RW, Bremner SA, Carrington J, Peacock J (2007). Quantitative Systematic Review of Short Term Associations between Ambient Air Pollution (Particulate Matter, Ozone, Nitrogen Dioxide, Sulphur Dioxide and Carbon Monoxide), and Mortality and Morbidity.. https://www.gov.uk/government/uploads/system/uploads/attachment_data/file/215975/dh_121202.pdf.

[r2] Armstrong B 2006 Models for the relationship between ambient temperature and daily mortality. Epidemiology 17 624 631, doi:10.1097/01.ede.0000239732.50999.8f 17028505

[r3] Braga AL, Zanobetti A, Schwartz J (2001). The lag structure between particulate air pollution and respiratory and cardiovascular deaths in 10 US cities.. J Occup Environ Med.

[r4] CETESB (Companhia Ambiental do Estado de São Paulo) (2014). Qualar – Qualidade do Ar [in Portuguese].. http://ar.cetesb.sp.gov.br/qualar/.

[r5] DAB (Departamento de Atenção Básica) (2015). Histórico de Cobertura da Saúde da Família [in Portuguese].. http://dab.saude.gov.br/portaldab/historico_cobertura_sf.php.

[r6] DATASUS (Departamento de Informática do SUS) (2014). Informações de Saúde (TABNET) – Estatísticas Vitais [in Portuguese].. http://www2.datasus.gov.br/DATASUS/index.php?area=0205&id=6937.

[r7] Filleul L Le Tertre A Baldi I Tessier JF 2004 Difference in the relation between daily mortality and air pollution among elderly and all-ages populations in southwestern France. Environ Res 94 249 253, doi:10.1016/S0013-9351(03)00080-X 15016591

[r8] Gasparrini A (2011). Distributed lag linear and non-linear models in R: the package dlnm.. J Stat Softw.

[r9] Gasparrini A 2014 Modeling exposure–lag–response associations with distributed lag non-linear models. Stat Med 33 881 899, doi:10.1002/sim.5963 24027094 PMC4098103

[r10] Gasparrini A Armstrong B Kenward MG 2010 Distributed lag non-linear models. Stat Med 29 2224 2234, doi:10.1002/sim.3940 20812303 PMC2998707

[r11] Gasparrini A Guo Y Hashizume M Lavigne E Zanobetti A Schwartz J 2015 Mortality risk attributable to high and low ambient temperature: a multicountry observational study. Lancet 386 369 375, doi:10.1016/S0140-6736(14)62114-0 26003380 PMC4521077

[r12] Goldberg MS Burnett RT Stieb DM Brophy JM Daskalopoulou SS Valois MF 2013 Associations between ambient air pollution and daily mortality among elderly persons in Montreal, Quebec. Sci Total Environ 463-464 931 942, doi:10.1016/j.scitotenv.2013.06.095 23872247

[r13] Goodman PG Dockery DW Clancy L 2004 Cause-specific mortality and the extended effects of particulate pollution and temperature exposure. Environ Health Perspect 112 179 185, doi:10.1289/ehp.6451 14754572 PMC1241827

[r14] IBGE (Instituto Brasileiro de Geografia e Estatística) (2015). Pesquisa Nacional de Saúde 2013: Acesso e Utilização dos Serviços de Saúde, acidentes e violências. Brasil, Grandes Regiões e Unidades da Federação [in Portuguese].. http://www.ibge.gov.br/home/estatistica/populacao/pns/2013_vol2/default.shtm.

[r15] ICEA (Instituto do Controle do Espaço Aéreo) (2014). Indicadores Meteorológicos–São Paulo [in Portuguese].. http://www.icea.gov.br/pesquisa/climatologia/sp.php.

[r16] IEMA (Instituto de Energia e Meio Ambiente) (2014). 1º Diagnóstico da Rede de Monitoramento da Qualidade do Ar no Brasil [in Portuguese].. http://www.energiaeambiente.org.br/wp-content/uploads/2015/08/1-diagnostico-da-rede-de-monitoramento-da-qualidade-do-ar-no-brasil.pdf.

[r17] INMET (Instituto Nacional de Meteorologia) (2014). BDMEP–Banco de Dados Meteorológicos para Ensino e Pesquisa [in Portuguese].. http://www.inmet.gov.br/portal/index.php?r=bdmep/bdmep.

[r18] Junger W, Ponce de Leon A (2012). mtsdi: multivariate time series data imputation (R package).. http://CRAN.R-project.org/package=mtsdi.

[r19] Martins LC Pereira LAA Lin CA Santos UP Prioli G, Luiz O do C, et al. 2006 The effects of air pollution on cardiovascular diseases: lag structures. Rev Saude Publica 40 677 683 17063245 10.1590/s0034-89102006000500018

[r20] O’Neill MS Bell ML Ranjit N Cifuentes LA Loomis D Gouveia N 2008 Air pollution and mortality in Latin America: the role of education. Epidemiology 19 810 819, doi:10.1097/EDE.0b013e3181816528 18633327

[r21] Roberts S Martin MA 2007 A distributed lag approach to fitting non-linear dose-response models in particulate matter air pollution time series investigations. Environ Res 104 193 200, doi:10.1016/j.envres.2007.01.009 17362914

[r22] Romieu I, Gouveia N, Cifuentes LA, de Leon AP, Junger W, Vera J (2012). Multicity study of air pollution and mortality in Latin America (the ESCALA study).. Res Rep Health Eff Inst.

[r23] Samoli E Stafoggia M Rodopoulou S Ostro B Declercq C Alessandrini E 2013 Associations between fine and coarse particles and mortality in Mediterranean cities: results from the MED-PARTICLES project. Environ Health Perspect 121 932 938, doi:10.1289/ehp.1206124 23687008 PMC3734494

[r24] Samoli E Zanobetti A Schwartz J Atkinson R LeTertre A Schindler C 2009 The temporal pattern of mortality responses to ambient ozone in the APHEA project. J Epidemiol Community Health 63 960 966, doi:10.1136/jech.2008.084012 19648130

[r25] Schwartz J (2000a). Harvesting and long term exposure effects in the relation between air pollution and mortality.. Am J Epidemiol.

[r26] Schwartz J (2000b). The distributed lag between air pollution and daily deaths.. Epidemiology.

[r27] SMSSP (Secretaria Municipal da Saúde da Prefeitura de São Paulo) (2014). PRO-AIM - Programa de Aprimoramento das Informações de Mortalidade [in Portuguese].. http://www.prefeitura.sp.gov.br/cidade/secretarias/saude/epidemiologia_e_informacao/mortalidade/index.php?p=29586.

[r28] Villeneuve PJ Burnett RT Shi Y Krewski D Goldberg MS Hertzman C 2003 A time-series study of air pollution, socioeconomic status, and mortality in Vancouver, Canada. J Expo Anal Environ Epidemiol 13 427 435, doi:10.1038/sj.jea.7500292 14603343

[r29] WHO (World Health Organization) (2006). *Air Quality Guidelines: Global Update 2005. Particulate Matter, Ozone, Nitrogen Dioxide and Sulfur Dioxide*.. http://www.euro.who.int/__data/assets/pdf_file/0005/78638/E90038.pdf.

[r30] WHO (2011). *Global Atlas on Cardiovascular Disease Prevention and Control*.. http://apps.who.int/iris/bitstream/10665/44701/1/9789241564373_eng.pdf?ua=1.

[r31] WHO (2013). *Review of Evidence on Health Aspects of Air Pollution— REVIHAAP Project. Technical Report*.. http://www.euro.who.int/__data/assets/pdf_file/0004/193108/REVIHAAP-Final-technical-report-final-version.pdf?ua=1.

[r32] Wood SN (2011). Fast stable restricted maximum likelihood and marginal likelihood estimation of semiparametric generalized linear models.. J R Stat Soc Series B Stat Methodol.

[r33] Zanobetti A Schwartz J 2008 Mortality displacement in the association of ozone with mortality: an analysis of 48 cities in the United States. Am J Respir Crit Care Med 177 184 189, doi:10.1164/rccm.200706-823OC 17932375

[r34] Zanobetti A, Schwartz J, Samoli E, Gryparis A, Touloumi G, Atkinson R (2002). The temporal pattern of mortality responses to air pollution: a multicity assessment of mortality displacement.. Epidemiology.

[r35] Zanobetti A Schwartz J Samoli E Gryparis A Touloumi G Peacock J 2003 The temporal pattern of respiratory and heart disease mortality in response to air pollution. Environ Health Perspect 111 1188 1193, doi:10.1289/ehp.5712 12842772 PMC1241573

[r36] Zanobetti A Wand MP Schwartz J Ryan LM 2000 Generalized additive distributed lag models: quantifying mortality displacement. Biostatistics 1 279 292, doi:10.1093/biostatistics/1.3.279 12933509

[r37] Zeger SL, Dominici F, Samet J (1999). Harvesting-resistant estimates of air pollution effects on mortality.. Epidemiology.

