# Supplementary material for: Air Pollution and Deaths among Elderly Residents of São Paulo, Brazil: An Analysis of Mortality Displacement
Source: Environ Health Perspect. 2016 Oct 7;125(3):349–54. doi: 10.1289/EHP98 (PMC5332200; doi:10.1289/EHP98)
Supplement: (333 KB) PDF [file EHP98.s001.acco.pdf]

Environ Health Perspect
DOI: 10.1289/EHP98

Note to readers with disabilities: *EHP* strives to ensure that all journal content is accessible to all readers. However, some figures and Supplemental Material published in *EHP* articles may not conform to 508 standards due to the complexity of the information being presented. If you need assistance accessing journal content, please contact ehponline@niehs.nih.gov. Our staff will work with you to assess and meet your accessibility needs within 3 working days.

Supplemental Material

Air Pollution and Deaths among Elderly Residents of São Paulo, Brazil: An Analysis of Mortality Displacement

Amine Farias Costa, Gerard Hoek, Bert Brunekreef, and Antônio CM Ponce de Leon

Table S1. Spearman correlations of PM10, CO and NO_2 concentrations among monitoring sites.

Table S2. Percent of missing data of PM10, CO and NO_2 among monitoring sites

Table S3. Details of models adjustment for trend, seasonality, temperature and relative humidity

Table S4. Cumulative percent change (95% confidence interval) in number of deaths associated with PM10 levels for different cumulative lag structures. Temporal trend sensitivity analysis

Table S5. Cumulative percent change (95% confidence interval) in number of deaths (all ages) associated with PM10 levels for different cumulative lag structures

Table S6. Cumulative percent change (95% confidence interval) in number of deaths associated with PM10 levels for different cumulative lag structures, adjusted by mean temperature until lag 10

Table S7. Comparison of single lag percent change (95% confidence interval) in number of total, circulatory, respiratory and cancer deaths among studies

Table S8. Comparison of cumulative percent change (95% confidence interval) by shorter lag structures in number of total, circulatory and respiratory deaths by 10 μg/m^3 increase in particulate air pollution among studies

Figure S1. Single lag percent change[a] in number of deaths associated with air pollutant levels of lags 0-30 days[b]. [a]Associated with a 10 µg/m^3 increase in PM10 and NO_2 and with a 1 ppm increase in CO. [b]Results from a Poisson generalized additive distributed lag model, constrained with a second degree polynomial, using single-day lag structures of lags 0-30 days for PM10, NO_2 and CO, adjusted by trend, seasonality, temperature, relative humidity, weekdays and holidays. The shadow area represents 95% CI.

References

Table S1. Spearman correlations of PM10, CO and NO_2 concentrations[a] among monitoring sites.

Sites	Cerqueira César	Ibirapuera	Parque Dom Pedro II
PM10			
Cerqueira César	1.00		
Ibirapuera	0.90	1.00	
Parque Dom Pedro II	0.90	0.90	1.00
CO			
Cerqueira César	1.00		
Ibirapuera	0.82	1.00	
Parque Dom Pedro II	0.84	0.80	1.00
NO_2			
Cerqueira César	1.00		-
Ibirapuera	0.80	1.00	-

[a]24-hour average for PM10 and NO_2, 8-hour maximum for CO.

Table S2. Percent of missing data of PM10, CO, NO_2 and O_3 among monitoring sites, before and after imputation methods.

Sites	Percent of missing data		
	Original data	After linear regressions	After *mtsdi* method
PM10			
Cerqueira César	10.6	0.43	0
Ibirapuera	5.5	1.12	0
Parque Dom Pedro II	18.1	0.43	0
CO			
Cerqueira César	3.2	0.23	0
Ibirapuera	6.3	0.23	0
Parque Dom Pedro II	19.1	0.34	0
NO_2			
Cerqueira César	23.8	1.60	0
Ibirapuera	16.2	5.23	0

Table S3. Details of models adjustment for trend, seasonality, temperature and relative humidity.

Deaths	Variables					
	Trend and seasonality		Temperature – Mean temperature		Relative humidity – Mean relative humidity	
	Spline	df/year (df after model fit)	Lag	df	Lag	df
Non-accidental	penalized	PM10: 6 (71.4)<br>NO_2: 6 (71.4)<br>CO: 6 (71.7)	3	3	3	2
Circulatory	penalized and unpenalized	PM10: 6 (70.8)<br>NO_2: 6 (72)<br>CO: 6 (72)	5	3	3	3
Cerebrovascular	unpenalized	4 (48)	5	3	3	3
Respiratory	unpenalized	5 (61)	3	3	3	2
Chronic respiratory	unpenalized	5 (61)	3	3	3	2
Cancer	unpenalized	4 (48)	5	2	3	2

Table S4. Cumulative percent change (95% confidence interval)[a] in number of deaths associated with PM10 levels for different cumulative lag structures[b]. Temporal trend sensitivity analysis.

Deaths (df/year)	Percent Change (95% CI)		
	Lag 0 to 10	Lag 0 to 20	Lag 0 to 30
Non-accidental (4)	0.91 (0.54, 1.28)	0.18 (-0.33, 0.69)	-0,59 (-1.25, 0.07)
Circulatory (5)	0.65 (0.06, 1.24)	-0.25 (-1.07, 0.57)	-1.09 (-2.15, -0.02)
Cerebrovascular (5)	2.30 (1.08, 3.54)	0.99 (-0.71, 2.72)	0.59 (-1.63, 2.87)
Respiratory (6)	3.24 (2.24, 4.26)	2.97 (1.53, 4.43)	2.76 (0.83, 4.72)
Chronic Respiratory (6)	3.07 (1.37, 4.80)	1.84 (-0.59, 4.32)	2.38 (-0.88, 5.74)
Cancer (5)	1.62 (0.75, 2.50)	1.72 (0.50, 2.96)	2.20 (0.58, 3.84)

[a]Associated with a 10 μg/m^3 increase in PM10. [b]Results from a Poisson generalized additive distributed lag model, constrained with a second degree polynomial, using cumulative lag structures of lags 0-10, 0-20 and 0-30 days for PM10, NO_2 and CO, adjusted by trend, seasonality, temperature, relative humidity, weekdays and holidays.

Table S5. Cumulative percent change (95% confidence interval)[a] in number of deaths (all ages) associated with PM10 levels for different cumulative lag structures[b].

Deaths	Percent Change (95% CI)		
	Lag 0 to 10	Lag 0 to 20	Lag 0 to 30
Non-accidental	1.07 (0.75, 1.39)	0.27 (-0.18, 0.72)	0.09 (-0.50, 0.69)
Circulatory	0.08 (-0.45, 0.61)	-1.12 (-1.86, -0.38)	-1.47 (-2.43, 0.50)
Cerebrovascular	1.75 (0.72, 2.79)	0.50 (-0.92, 1.93)	0.12 (-1.70, 1.98)
Respiratory	3.07 (2.21, 3.94)	3.07 (1.85, 4.31)	2.97 (1.35, 4.62)
Chronic Respiratory	2.87 (1.36, 4.41)	1.97 (-0.15, 4.14)	2.34 (-0.49, 5.25)
Cancer	1.48 (0.78, 2.18)	1.33 (0.37, 2.30)	1.90 (0.65, 3.16)

[a]Associated with a 10 µg/m^3 increase in PM10. [b]Results from a Poisson generalized additive distributed lag model, constrained with a second degree polynomial, using cumulative lag structures of lags 0-10, 0-20 and 0-30 days for PM10, NO_2 and CO, adjusted by trend, seasonality, temperature, relative humidity, weekdays and holidays.

Table S6. Cumulative percent change (95% confidence interval)[a] in number of deaths associated with PM10 levels for different cumulative lag structures, adjusted by mean temperature until lag 10[b].

Deaths	Percent Change (95% CI)		
	Lag 0 to 10	Lag 0 to 20	Lag 0 to 30
Non-accidental	1.28 (0.81, 1.75)	1.07 (0.42, 1.73)	0.49 (-0.34, 1.35)
Circulatory	1.04 (0.31, 1.78)	0.50 (-0.53, 1.53)	-0.45 (-1.75, 0.87)
Cerebrovascular	1.38 (0.03, 2.75)	0.33 (-1.50, 2.19)	-0.23 (-2.53, 2.12)
Respiratory	1.99 (0.86, 3.12)	2.05 (0.49, 3.63)	1.70 (-0.28, 3.73)
Chronic Respiratory	1.73 (-0.17, 3.66)	0.77 (-1.83, 3.44)	1.22 (-2.12, 4.68)
Cancer	1.01 (0.13, 1.89)	1.37 (0.21, 2.61)	1.69 (0.22, 3.20)

[a]Associated with a 10 µg/m^3 increase in PM10. [b]Results from a Poisson generalized additive distributed lag model, constrained with a second degree polynomial, using cumulative lag structures of lags 0-10, 0-20 and 0-30 days for PM10, NO_2 and CO, adjusted by trend, seasonality, temperature (cumulative lag structures of lags 0-10 days with exposure-response and lag-response curves according to Gasparrini et al. (2015)), relative humidity, weekdays and holidays.

Table S7. Comparison of single lag[a] percent change (95% confidence interval)[b] in number of total, circulatory, respiratory and cancer deaths[c] among studies.

Deaths	Percent change (95% CI)		
	PM10	NO2	CO
**Non-accidental**			
São Paulo (this study)	0.54 (0.36,0.72)	0.58 (0.38, 0.79)	1.07 (0.68, 1.47)
ESCALA (Romieu et al. 2012)			
São Paulo	0.49 (0.30, 0.70)	NA	NA
Rio de Janeiro	0.57 (0.20, 0.90)	NA	NA
Meta-analysis (Anderson et al. 2007)	0.47 (0.35, 0.58)	0.86 (0.50, 1.22)	1.56 (-0.37, 3.52)
APHEA (Aga et al. 2003; Samoli et al. 2006, 2007)	0.74 (0.52, 0.95)	0.30 (0.22, 0.38)	1.20 (0.63, 1.77)
APHENA (Katsouyanni et al. 2009)			
Canada	1.10 (0.64, 1.60)	NA	NA
USA	0.62 (0.39, 0.86)	NA	NA
Europe	0.45 (0.29, 0.61)	NA	NA
PAPA (Wong et al. 2008)	0.55 (0.26, 0.85)	1.23 (0.84,1.62)	NA
Meta-analysis (Mills et al. 2015)	NA	0.71 (0.43, 1.00)	NA
**Circulatory**			
São Paulo (this study)	0.40 (0.07, 0.73)	0.42 (0.11, 0.74)	0.84 (0.23, 1.45)
ESCALA (Romieu et al. 2012)			
São Paulo	0.46 (0.00, 1.00)	NA	NA
Rio de Janeiro	1.09 (0.40, 2.00)	NA	NA
Meta-analysis (Anderson et al. 2007)	0.50 (0.31, 0.69)	0.34 (0.19, 0.48)	1.11 (0.48, 1.74)
APHEA (Samoli et al. 2006, 2007; Zanobetti et al. 2003)	0.69 (0.31, 1.08)	0.40 (0.29, 0.52)	0.81 (0.36; 1.26)
APHENA (Katsouyanni et al. 2009)			
Canada	1.80 (0.75, 2.80)	NA	NA
USA	1.01 (0.60, 1.42)	NA	NA
Europe	0.74 (0.45, 1.03)	NA	NA
PAPA (Wong et al. 2008)	0.58 (0.22, 0.93)	1.36 (0.89,1.82)	NA
Meta-analysis (Mills et al. 2015)	NA	0.88 (0.63, 1.13)	NA

Table S7 (continued)

**Respiratory**			
São Paulo (this study)	0.98 (0.41, 1.56)	0.67 (0.10, 1.24)	2.54 (1.47, 3.63)
ESCALA (Romieu et al. 2012)			
São Paulo	0.80 (0.10, 1.60)	NA	NA
Rio de Janeiro	1.50 (0.00, 2.70)	NA	NA
Meta-analysis (Anderson et al. 2007)	1.33 (0.75, 1.91)	0.45 (0.21, 0.69)	2.65 (1.27,4.05)
APHEA (Samoli et al. 2006, 2007; Zanobetti et al. 2003)	0.74 (-0.17, 1.66)	0.38 (0.17, 0.58)	NA
APHENA (Katsouyanni et al. 2009)			
Canada	-0.36 (-2.00, 1.30)	NA	NA
USA	1.34 (0.73, 1.96)	NA	NA
Europe	0.62 (0.28, 0.96)	NA	NA
PAPA (Wong et al. 2008)	0.62 (0.22, 1.02)	1.48 (0.68, 2.28)	NA
Meta-analysis (Mills et al. 2015)	NA	1.09 (0.75, 1.42)	NA
**Cerebrovascular**			
São Paulo (this study)	1.44 (0.74, 2.15)	1.32 (0.63, 2.02)	2.39 (1.08, 3.71)
ESCALA (Romieu et al. 2012)			
São Paulo	0.56 (-0.30, 1.40)	NA	NA
Rio de Janeiro	1.43 (0.10, 2.80)	NA	NA
Meta-analysis (Anderson et al. 2007)	0.55 (0.15,0.95)	0.94 (0.34,1.55)	3.90 (1.72,6.13)
Meta-analysis (Mills et al. 2015)	NA	1.35 (0.74, 1.97)	NA
Meta-analysis (Shah et al. 2015)	0.30 (0.20, 0.40)	1.60 (0.70, 2.30)	5.40 (0.10, 10.80)
**Chronic Respiratory**			
São Paulo (this study)	0.56 (-0.29, 1.41)	1.21 (0.34, 2.08)	2.19 (0.41, 4.01)
ESCALA (Romieu et al. 2012)			
São Paulo	0.52 (-0.50, 1.50)	NA	NA
Rio de Janeiro	0.47 (-1.70, 2.50)	NA	NA
Meta-analysis (Anderson et al. 2007)	0.53 (-0.22,1.28)	1.92 (1.07,2.78)	5.36 (2.64,8.16)
Meta-analysis (Mills et al. 2015)	NA	1.11 (0.72, 1.50)	NA
Meta-analysis (Shah et al. 2015)	2.00 (1.00, 3.00)	NA	NA

Table S7 (continued)

**Cancer**			
São Paulo (this study)	0.71 (0.27, 1.15)	0.93 (0.48, 1.38)	0.16 (-0.83, 1.16)
Vancouver (Villeneuve et al. 2003)	2.30 (-4.60, 9.60)	3.50 (-2.30, 9.70)	-1.60 (-5.60, 2.40)
Montreal (Goldberg et al. 2013)	NA	1.92 (0.14, 3.74)	0.79 (-0.83, 2.43)

ESCALA: Estudio de Salud y Contaminación del Aire en Latinoamérica. APHEA: Air Pollution and Health: a European Approach. APHENA: Air Pollution and Health: A European and North American Approach. PAPA: Public Health and Air Pollution in Asia. NA: not available.

[a]Lag 0, 1, 2 or mean of lags 0 and 1. [b]Associated with an interquatile range in Villeneuve et al. (2003) and and with a 10 μg/m^3 increase in PM10 and NO_2 and with a 1 ppm increase in CO for other studies. [c]Elderly deaths whenever possible.

Table S8. Comparison of cumulative percent change (95% confidence interval) by shorter lag structures in number of total, circulatory and respiratory deaths by 10 μg/m^3 increase in particulate air pollution among studies.

**Deaths**	Age group	Air pollutant	Period	Percent change (CI)
**Non-accidental**				
São Paulo[a] (this study)	Elderly	PM10	Lag 0 to 3	0.85 (0.62, 1.08)
São Paulo (Romieu et al. 2012)[a]	All ages	PM10	Lag 0 to 3	0.79 (0.64, 0.94)
São Paulo[a] (this study)	Elderly	PM10	Lag 0 to 5	1.08 (0.79, 1.37)
São Paulo (O'Neill et al. 2008)[b]	Elderly	PM10	Lag 0 to 5	1.17 (0.50, 1.85)
10 USA cities (Schwartz 2000)[a]	Elderly	PM2.5	Lag 0 to 5	1.41 (1.16, 1.67)
12 European cities (Samoli et al. 2013)[a]	All ages	PM10	Lag 0 to 5	0.28 (–0.14, 0.71)
**Circulatory deaths**				
São Paulo[a] (this study)	Elderly	PM10	Lag 0 to 3	0.50 (0.14, 0.85)
São Paulo (Romieu et al. 2012)[a]	Elderly	PM10	Lag 0 to 3	0.81 (0.50, 1.12)
São Paulo[a] (this study)	Elderly	PM10	Lag 0 to 5	0.64 (0.19, 1.09)
12 European cities (Samoli et al. 2013)[a]	All ages	PM10	Lag 0 to 5	0.54 (0.09, 0.99)
São Paulo[a] (this study)	Elderly	PM10	Lag 0 to 6	0.68 (0.19, 1.17)
10 USA cities (Braga et al. 2001)[b]	All ages	PM10	7-day effect	1.00 (0.60, 1.40)
**Respiratory deaths**				
São Paulo[a] (this study)	Elderly	PM10	Lag 0 to 3	2.41 (1.84, 2.99)
São Paulo (Romieu et al. 2012)[a]	Elderly	PM10	Lag 0 to 3	1.52 (1.00, 2.03)
São Paulo[a] (this study)	Elderly	PM10	Lag 0 to 5	3.02 (2.30, 3.75)
12 European cities (Samoli et al. 2013)[a]	All ages	PM10	Lag 0 to 5	1.12 (0.29, 1.95)
**Chronic respiratory deaths**				
São Paulo[a] (this study)	Elderly	PM10	Lag 0 to 3	2.26 (1.3, 3.24)
São Paulo (Romieu et al. 2012)[a]	Elderly	PM10	Lag 0 to 3	1.81 (0.97, 2.65)
São Paulo[a] (this study)	Elderly	PM10	Lag 0 to 6	2.96 (1.64, 4.29)
10 USA cities (Braga et al. 2001)[b]	All ages	PM10	7-day effect	1.70 (0.10, 3.30)

SE: standard error.
[a]Distributed lag model constrained with polynomial structure. [b]Unconstrained distributed lag model.

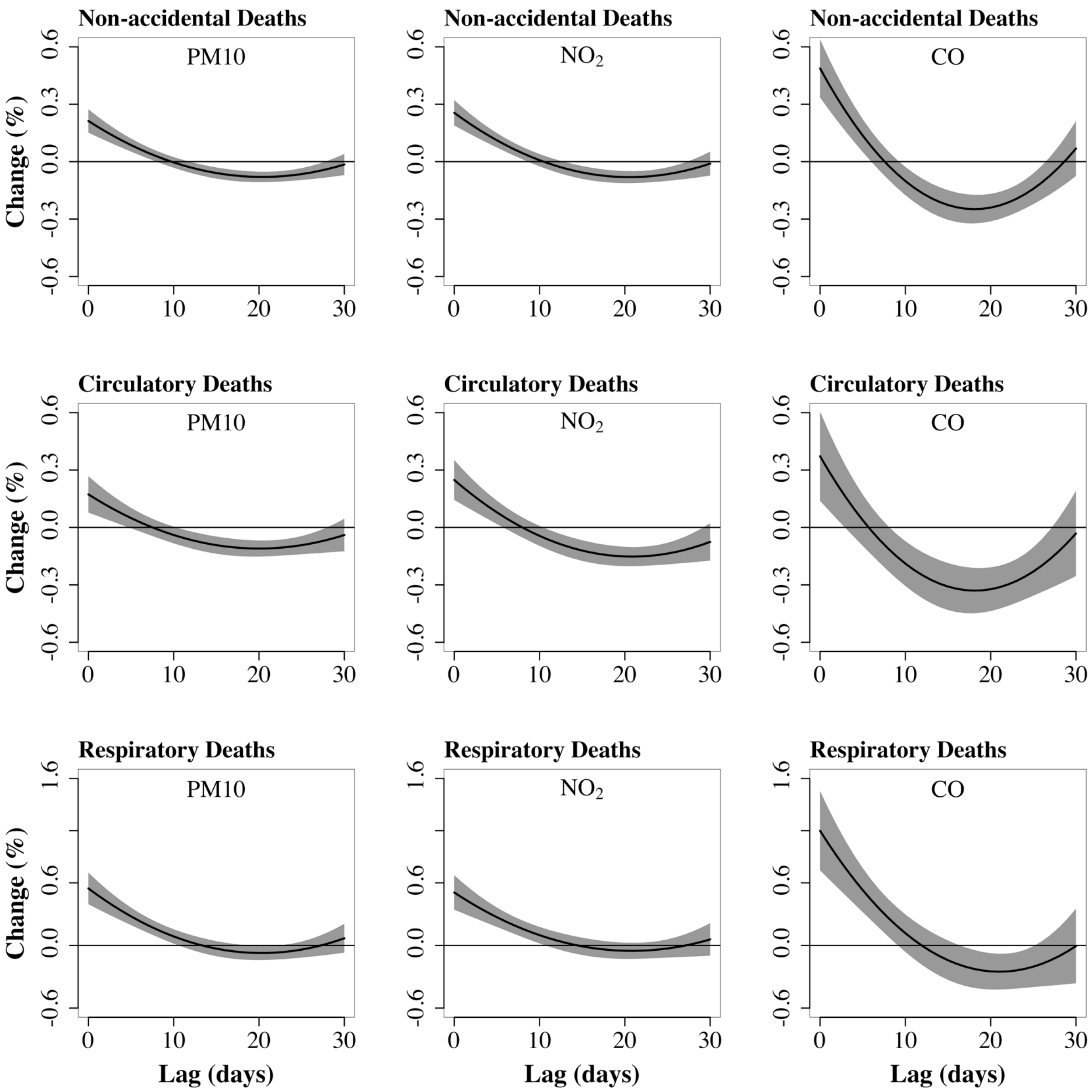

Figure S1. Single lag percent change[a] in number of deaths associated with air pollutant levels of lags 0-30 days[b].

[a]Associated with a 10 μg/m^3 increase in PM10 and NO_2 and with a 1 ppm increase in CO. [b]Results from a Poisson generalized additive distributed lag model, constrained with a second degree polynomial, using single-day lag structures of lags 0-30 days for PM10, NO_2 and CO, adjusted by trend, seasonality, temperature, relative humidity, weekdays and holidays. The shadow area represents 95% CI.

References

Aga E, Samoli E, Touloumi G, Anderson HR, Cadum E, Forsberg B, et al. 2003. Short-term effects of ambient particles on mortality in the elderly: results from 28 cities in the APHEA2 project. Eur Respir J Suppl 40:28–33.

Anderson H, Atkinson R, Bremner S, Carrington J, Peacock J. 2007. Quantitative systematic review of short term associations between ambient air pollution (particulate matter, ozone, nitrogen dioxide, sulphur dioxide and carbon monoxide), and mortality and morbidity. London:Department of Health. Available: https://www.gov.uk/government/publications/quantitative-systematic-review-of-short-term-associations-between-ambient-air-pollution-particulate-matter-ozone-nitrogen-dioxide-sulphur-dioxide-and-carbon-monoxide-and-mortality-and-morbidity [accessed 10 June 2015].

Braga AL, Zanobetti A, Schwartz J. 2001. The lag structure between particulate air pollution and respiratory and cardiovascular deaths in 10 US cities. J Occup Environ Med 43: 927–933.

Gasparrini A, Guo Y, Hashizume M, Lavigne E, Zanobetti A, Schwartz J, et al. 2015. Mortality risk attributable to high and low ambient temperature: a multicountry observational study. Lancet 386:369–375; doi:10.1016/S0140-6736(14)62114-0.

Goldberg MS, Burnett RT, Stieb DM, Brophy JM, Daskalopoulou SS, Valois M-F, et al. 2013. Associations between ambient air pollution and daily mortality among elderly persons in Montreal, Quebec. Sci Total Environ 463-464:931–942; doi:10.1016/j.scitotenv.2013.06.095.

Katsouyanni K, Samet JM, Anderson HR, Atkinson R, Le Tertre A, Medina S, et al. 2009. Air pollution and health: a European and North American approach (APHENA). Res Rep Health Eff Inst 142:5–90.

Mills IC, Atkinson RW, Kang S, Walton H, Anderson HR. 2015. Quantitative systematic review of the associations between short-term exposure to nitrogen dioxide and mortality and hospital admissions. BMJ Open 5:e006946; doi:10.1136/bmjopen-2014-006946.

O'Neill MS, Bell ML, Ranjit N, Cifuentes LA, Loomis D, Gouveia N, et al. 2008. Air pollution and mortality in Latin America: the role of education. Epidemiology 19:810–819; doi:10.1097/EDE.0b013e3181816528.

Romieu I, Gouveia N, Cifuentes LA, de Leon AP, Junger W, Vera J, et al. 2012. Multicity study of air pollution and mortality in Latin America (the ESCALA study). Res Rep Health Eff Inst.171:5–86.

Samoli E, Aga E, Touloumi G, Nisiotis K, Forsberg B, Lefranc A, et al. 2006. Short-term effects of nitrogen dioxide on mortality: an analysis within the APHEA project. Eur Respir J 27:1129–1138; doi:10.1183/09031936.06.00143905.

Samoli E, Stafoggia M, Rodopoulou S, Ostro B, Declercq C, Alessandrini E, et al. 2013. Associations between fine and coarse particles and mortality in Mediterranean cities:

results from the MED-PARTICLES project. Environ Health Perspect 121:932–938; doi:10.1289/ehp.1206124.

Samoli E, Touloumi G, Schwartz J, Anderson HR, Schindler C, Forsberg B, et al. 2007. Short-term effects of carbon monoxide on mortality: an analysis within the APHEA project. Environ Health Perspect 115:1578–1583; doi:10.1289/ehp.10375.

Schwartz J. 2000. The distributed lag between air pollution and daily deaths. Epidemiology 11:320–326.

Shah ASV, Lee KK, McAllister DA, Hunter A, Nair H, Whiteley W, et al. 2015. Short term exposure to air pollution and stroke: systematic review and meta-analysis. BMJ 350: h1295; doi: 10.1136/bmj.h1295.

Villeneuve PJ, Burnett RT, Shi Y, Krewski D, Goldberg MS, Hertzman C, et al. 2003. A time-series study of air pollution, socioeconomic status, and mortality in Vancouver, Canada. J Expo Anal Environ Epidemiol 13:427–435; doi:10.1038/sj.jea.7500292.

Wong C-M, Vichit-Vadakan N, Kan H, Qian Z. 2008. Public Health and Air Pollution in Asia (PAPA): a multicity study of short-term effects of air pollution on mortality. Environ Health Perspect 116:1195–1202; doi:10.1289/ehp.11257.

Zanobetti A, Schwartz J, Samoli E, Gryparis A, Touloumi G, Peacock J, et al. 2003. The temporal pattern of respiratory and heart disease mortality in response to air pollution. Environ Health Perspect 111:1188–1193.
